# Vaccination of mice with a recombinant novel cathepsin B inhibits *Trichinella spiralis* development, reduces the fecundity and worm burden

**DOI:** 10.1186/s13071-019-3833-9

**Published:** 2019-12-11

**Authors:** Jing Cui, Yue Han, Xin Yue, Fang Liu, Yan Yan Song, Shu Wei Yan, Jun Jun Lei, Xi Zhang, Peng Jiang, Zhong Quan Wang

**Affiliations:** Department of Parasitology, Medical College, Zhengzhou University, Zhengzhou, 450052 China

**Keywords:** *Trichinella spiralis*, Trichinellosis, Cathepsin B, Vaccination, Immune protection

## Abstract

**Background:**

*Trichinella spiralis* is a major zoonotic tissue-dwelling nematode, which is a public health concern and a serious hazard to animal food safety. It is necessary to exploit an anti-*Trichinella* vaccine to interrupt the transmission of *Trichinella* infection among animals and from animals to humans. The purpose of the present study was to characterize the novel *T. spiralis* cathepsin B (TsCB) and to evaluate the immune protection elicited by immunization with recombinant TsCB (rTsCB).

**Methods:**

The complete cDNA sequences of the TsCB gene were cloned, expressed and purified. The antigenicity of rTsCB was investigated by western blot analysis and ELISA. Transcription and expression of TsCB at various *T. spiralis* life-cycle stages were analyzed by RT-PCR and indirect immunofluorescent assay (IIFA). The mice were subcutaneously immunized with rTsCB, and serum level of TsCB-specific IgG (IgG1 and IgG2a) and IgE antibodies were assayed by ELISA. Immune protection elicited by vaccination with rTsCB was investigated.

**Results:**

The TsCB was transcribed and expressed in four *T. spiralis* life-cycle stages (adult worm, AW; newborn larvae, NBL; muscle larvae, ML; and intestinal infective L1 larvae), it was primarily located in the cuticle and stichosome of the parasitic nematode. Vaccination of mice with rTsCB produced a prominent antibody response (high level of specific IgG and IgE) and immune protection, as demonstrated by a 52.81% AW burden reduction of intestines at six days post-infection (dpi) and a 50.90% ML burden reduction of muscles at 35 dpi after oral larva challenge. The TsCB-specific antibody response elicited by immunization with rTsCB also impeded intestinal worm growth and decreased the female fecundity.

**Conclusions:**

TsCB might be considered as a novel potential molecular target to develop vaccines against *T. spiralis* infection.

## Background

*Trichinella spiralis* is an important zoonotic tissue-dwelling nematode, the largest intracellular parasite which infects more than 150 different kinds of mammals and humans around the world [1]. *Trichinella spiralis* infection in humans is mainly resulting from ingesting raw or semi-raw meat or meat products infected with the encapsulated muscle larvae (ML) of this nematode. In the Chinese mainland, 14 trichinellosis outbreaks due to infected pork from domestic pigs and wild boar were documented during 2004–2009 [2]. Swine pork is the major infectious source of human *Trichinella* infection in developing countries and areas [3–5]. Infections with *Trichinella* spp. are not merely a public health concern but also a severe hazard to animal food safety [6, 7]. It is difficult to eradicate *Trichinella* spp. infection in animals as preventive anti-*Trichinella* vaccines are not currently available. [8, 9]. Screening and identification of *Trichinella* spp. invasion-related proteins is recommended to help identify novel candidate targets for a vaccine against *Trichinella* infection [10].

After being eaten, *T. spiralis* ML encapsulate in the skeletal muscles and are released from their capsules in the stomach, where they develop into intestinal infective L1 larvae (IIL1) within the intestines. The IIL1 larvae intrude into enteral epithelia and continue to grow into adult worms (AW) by molting four times [11, 12]. Female adults give birth to newborn larvae (NBL), which pass into the bloodstream, penetrate into the skeletal muscles and encapsulate to accomplish the life-cycle [13]. The intestinal epithelial invasion by IIL1 larvae is the first infection, but the invasion mechanism is not clear. As intestinal epithelia are the preferential natural barrier against larval invasion, and the major site for host-*T. spiralis* interaction [14, 15], identification of IIL1 invasive proteins will be valuable to understand invasion mechanisms of the parasite and develop vaccines against *T. spiralis* intestinal invasive worms [16, 17].

Cathepsin B is one member of the cysteine protease family, which plays an important function in worm invading, migrating, molting and immune escape [18, 19]. Cysteine proteases have been identified in excretion/secretion (ES) products or somatic proteins of *T. spiralis* ML and AW [20, 21]. When *T. spiralis* IIL1 larvae were inoculated onto an enteral epithelium cell monolayer, the IIL1 larvae penetrated the monolayer and expressed additional cysteine proteases which were found to be highly expressed at the IIL1 stage [22]. It might participate in IIL1 intrusion of the enteral epithelium during *Trichinella* infection [23–25].

In the present study, a novel cathepsin B gene of *T. spiralis* (TsCB, GenBank: XP_003379650.1) was obtained from the *T. spiralis* draft genome [26], cloned and expressed. The TsCB were characterized and the protective immunity triggered by rTsCB immunization were investigated in a mouse model.

## Methods

### Worm maintenance and experimental animals

*Trichinella spiralis* (ISS534) isolated from a domestic pig in central China was maintained in mice by serial passage in our laboratory [27]. Six-week-old female BALB/c mice were provided by the animal centre at Zhengzhou University.

### Worm collection and antigen preparation

The ML were recovered by artificially digesting *T. spiralis*-infected mouse muscles at 35 days post-infection (dpi) [28, 29]. The IIL1 were isolated from mouse intestine at 6 hpi [30], and the AW were collected from mouse intestine on days 3 and 6 after infection [31]. After washes with sterile PBS, the day 6 AW were cultured in RPMI-1640 medium (Gibco, Auckland, New Zealand) containing 10% fetal bovine serum (50 female worms/ml), and the newborn larvae (NBL) were recovered 24 h following culture [32]. The soluble proteins of ML, IIL1, AW and NBL, and the ML ES proteins were prepared as previously reported [33, 34].

### Bioinformatics analysis of TsCB

The complete cDNA sequence of the TsCB gene was acquired from GenBank (GenBank: XP_003379650.1). The characteristics of TsCB gene sequences were analyzed on the Expasy website (http://web.expasy.org/protparam) as previously reported [35, 36]. PyMOL and CN3D software was used to predict the tertiary structure and functional sites of TsCB protein [37]. The cathepsin B sequences from *Trichinella* spp. and other organisms were retrieved from the GenBank database as follows: *T. nativa* (KRZ52829.1); *T. murrelli* (KRX41017.1); *Trichinella* sp. T6 (KRX78271.1); *Trichinella* sp. T8 (KRZ85246.1); *T. britovi* (KRY52058.1); *T. nelsoni* (KRX21942.1); *T. pseudospiralis* (KRY81987.1); *Trichuris suis* (KFD57259.1); *Trichuris trichiura* (CDW59512.1); *Necator americanus* (CAB53367.1); *Haemonchus contortus* (AAC05262.1); *Ascaris suum* (AAB40605.1); *Clonorchis sinensis* (ABM47070.1); *Schistosoma mansoni* (CAC85211.2); *S. japonicum* (CAA50305.1); *Hymenolepis microstoma* (CDS27962.1); *Homo sapiens* (NP_001899.1); and *Mus musculus* (EDL36070.1). The multiple alignment of the TsCB sequences with the cathepsin B (CB) homologues of other organisms was conducted using Clustal X [38]. A phylogenetic tree of these CB sequences was generated using the maximum parsimony (MP) method as described by Sun et al. [39].

### RT-PCR quantification of TsCB transcript levels

Total RNA was isolated with Trizol reagent (Invitrogen, Carlsbad, CA, USA) from worms of the different life-cycle stages (ML, IIL1, 3 days AW and NBL). TsCB transcript level at each stage was quantified by RT-PCR as reported [40]. *Trichinella spiralis* glyceraldehyde-3-phosphate dehydrogenase (GAPDH, GenBank: AF452239) was amplified as a housekeeping gene [41]. PBS was utilized as a negative control in all PCR amplification.

### Cloning and expression of rTsCB

Total RNA from the ML was extracted using Trizol (Invitrogen). The complete TsCB sequences were amplified by PCR with specific primers incorporating the restriction enzyme sites *Bam*HI and *Pst*I (restriction sites underlined: 5′-GCG GAT CCA TTC CTT TTG GTT CCA GA-3′; 5′-AGC TGC AGT CAC GTT GGC TTC TTG TAC-3′). The PCR product was cloned into the pQE-80L (Novagen, La Jolla, CA, USA), then the recombinant plasmid pQE-80L/TsCB was transformed into *Escherichia coli* BL21 (DE3) (Novagen). Expression of rTsCB was induced by 1 mM IPTG at 37 °C for 6 h [17] and subsequently purified using Ni-NTA-Sefinose resin (Sangon Biotech Co., Shanghai, China) [42, 43]. The rTsCB concentration was assayed and analyzed by SDS-PAGE at 120 V for 1.5 h [10].

### Immunization of mice and analysis of antibody responses to rTsCB

Sixty mice were divided into three groups of equal size (20 mice/group). Each mouse was subcutaneously vaccinated with 20 µg rTsCB emulsified with ISA 201 adjuvant (Seppic, Paris, France). Vaccination was repeated three times at 2-week intervals using the same dose of rTsCB and ISA 201. Control groups received only ISA 201 or PBS using the same vaccination procedure [44]. Individual serum samples were collected before vaccination and on weeks 2, 4, 6 and 8 after vaccination [45].

Specific antibody responses to rTsCB (total IgG, IgG1 as well IgG2a) in immunized mice were measured by ELISA two weeks after each vaccination [37, 46]. The IgE response was also assayed by indirect ELISA using rTsCB as the coating antigen. The ELISA plate was coated using 2 μg/ml rTsCB at 4 °C overnight. After washing, the plate was blocked using 5% skimmed milk in PBST, then probed with mouse immune sera (1:100) at 37 °C for 1 h. Goat anti-mouse IgG (IgG1 and IgG2a)-HRP conjugates (1:5000; Sigma-Aldrich, St. Louis, MO, USA) or Goat anti-mouse IgE-HRP conjugate (1:2500; Southern Biotech, Tuscaloosa, AL, USA) were added and incubated for 1 h at 37 °C. Detection was performed by adding the substrate OPD (Sigma-Aldrich, St. Louis, MO, USA) with 30% H_2_O_2_ for 20 min and terminated using 2M H_2_SO_4_ [47]. The absorbance at 492 nm was measured using a microplate reader (Tecan, Schweiz, AG, Switzerland) [48, 49].

### Western blot analysis

Samples contained various proteins: somatic proteins of ML, IIL1, AW and NBL, ML ES antigens, and rTsCB (8 μg protein/lane). The protein was separated using SDS-PAGE with a 12% resolving gel [33, 50]; the gel was then transferred to a nitrocellulose membrane (Merck Millipore, Billerica, MA, USA) [48]. The membrane was cut into strips, blocked using 5% skimmed milk in TBST. After three washes in TBST, the strips were probed using 1:100 dilution of three different mouse sera (anti-rTsCB serum, mouse infection serum, and pre-immune normal mouse serum) for 1 h at 37 °C. The blots were washed with TBST then incubated with anti-mouse IgG HRP-conjugate (1:10,000; Southern Biotech) at 37 °C for 1 h. Detection was achieved using 3, 3′-diaminobenzidine tetrahydrochloride (DAB; Sigma-Aldrich) [15, 51].

### Indirect immunofluorescence assay (IIFA)

Expression and tissue localization of natural TsCB in the nematode were investigated using IIFA with anti-rTsCB serum [42, 52]. Paraffin sections (3 µm thick) of the different worm life-cycle stages were used to examine TsCB expression and tissue localization in *T. spiralis*. Each section was blocked with 5% normal goat serum (Sangon, Shanghai, China), and probed at 37 °C for 1 h with three different sera (1:10; anti-rTsCB serum, mouse infection serum and pre-immune serum). Following three washes with PBS, the sections were stained at 37 °C for 1 h using FITC-conjugated anti-mouse IgG (1:100; Santa Cruz, USA). Sections were washed as previously reported and examined using fluorescent microscopy (Olympus, Tokyo, Japan) [49, 53].

### Challenge experiment

To investigate the immune protection offered by vaccination with rTsCB, all mice were infected orally with 300 *T. spiralis* ML at two weeks after the final boost. Intestinal adults were collected from 10 mice at 6 dpi [54], and muscle larvae at 35 dpi were obtained by artificially digesting the carcasses of the remaining 10 mice [55]. Immune protection was ascertained as worm reduction of enteral adults and larvae per gram (LPG) of skeletal muscles of immunized groups compared to those of the PBS control group [8, 56, 57].

### Statistical analysis

All statistical analysis was conducted using SPSS for Windows, version 22.0 (SPSS Inc., Chicago, IL, USA). The values are presented as the mean ± standard deviation (SD). Difference among various groups was analyzed using a Student’s t-test or one-way ANOVA. *P *< 0.05 was regarded as a level for statistical significance.

## Results

### Bioinformatics analysis of TsCB sequence

**Fig. 1:**
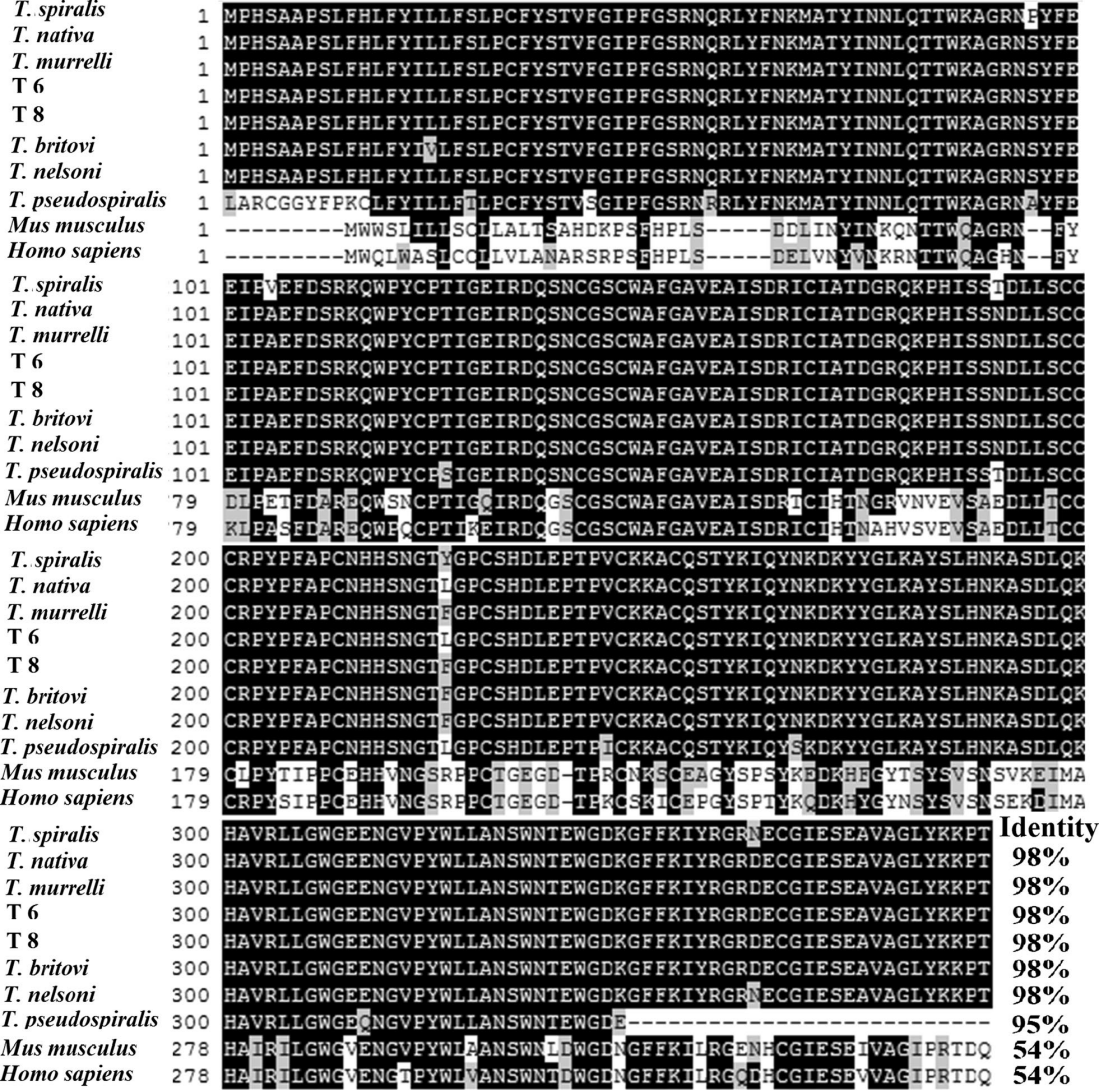
Sequence alignment of TsCB with cathepsin B from *Trichinella* spp. and other species. Sequence alignment was conducted in Clustal X and shown using BOXSHADE. Black shade indicates that residues were the same as TsCB; conservative substitutions are marked in grey. The percentage identity with TsCB is shown

Bioinformatics analyses revealed that the full-length TsCB sequence was 1071 bp, encoding a protein of 356 amino acids, with 40.23 kDa and 7.86 isoelectric point (pI). Analyses with Signal P 4.1 and TMHMM Server indicated that the signal peptide was located at 1–29 aa, TsCB had 7 α-helixes and 13 β-strands, and a transmembrane domain was located outside the cell membrane. Subcellular localization of TsCB was present in mitochondria (2%), periplasm (94.9%) and cytoplasm (6.7%), respectively. The homology comparison of TsCB sequences with those of other *Trichinella* species or genotypes are shown in Fig. 1. TsCB amino acid sequence had 98% identity with cathepsin B of *T. nativa*, *T. murrelli*, T6, T8, *T. britovi* and *T. nelsoni*, and 95% identity with *T. pseudospiralis.*

**Fig. 2:**
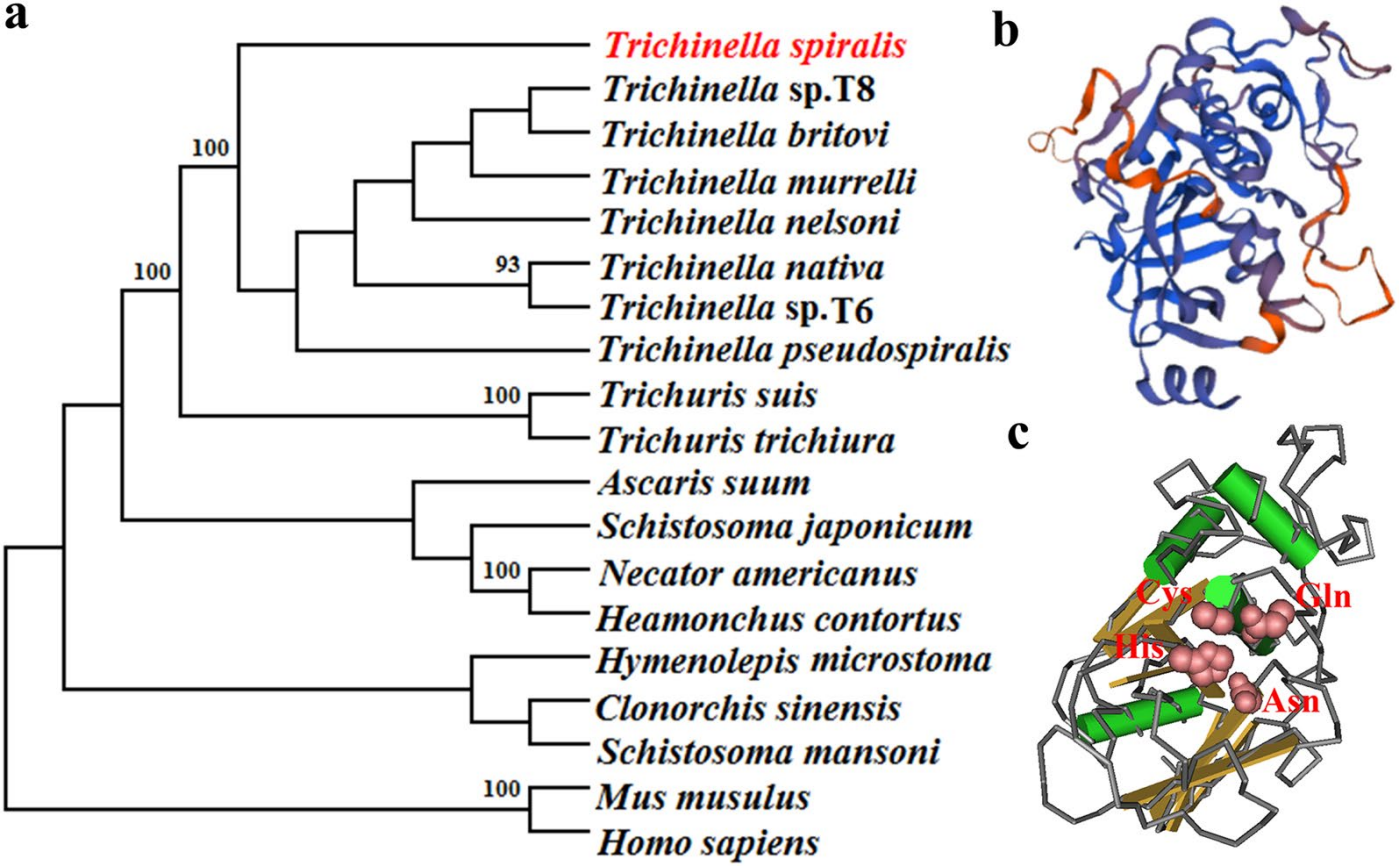
Phylogenetic trees of cathepsin B of 19 organisms estimated with the MP method (**a**) and the predicted 3-dimensional structure of *T. spiralis* cathepsin B (**b, c**). **b** The predicted 3-dimensional structure of TsCB contains 7 α-helixes (red) and 13 β-strand (blue). **c** Functional domain carrying catalytic reactive sites consisted of Gln124, Cys155, Asn305, and Gly328 residues, formed a functional domain. The TsCB active sites are highlighted in red

Phylogenetic analysis of TsCB with cathepsin B from other species is shown in Fig. 2a. The phylogenetic tree generated using the MP method verified a monophyletic group of the above-mentioned 7 species/gene types within the genus *Trichinella*. *Trichinella spiralis* has a close evolutionary relationship with encapsulated and non-encapsulated *Trichinella* species, and is more closely related to nematode cathepsin B than that from other species.

The SMART analysis results revealed that there was a functional domain (between positions 102–351 aa) of peptidase_C1A. In a 3-dimensional model, TsCB had the catalytic active sites, which were composed of Gln124, Cys130, His300 and Asn320 residues, forming a pocket-shaped functional domain carrying substrate binding sites (Fig. 2c).

### RT-PCR analysis of TsCB transcription

**Fig. 3:**
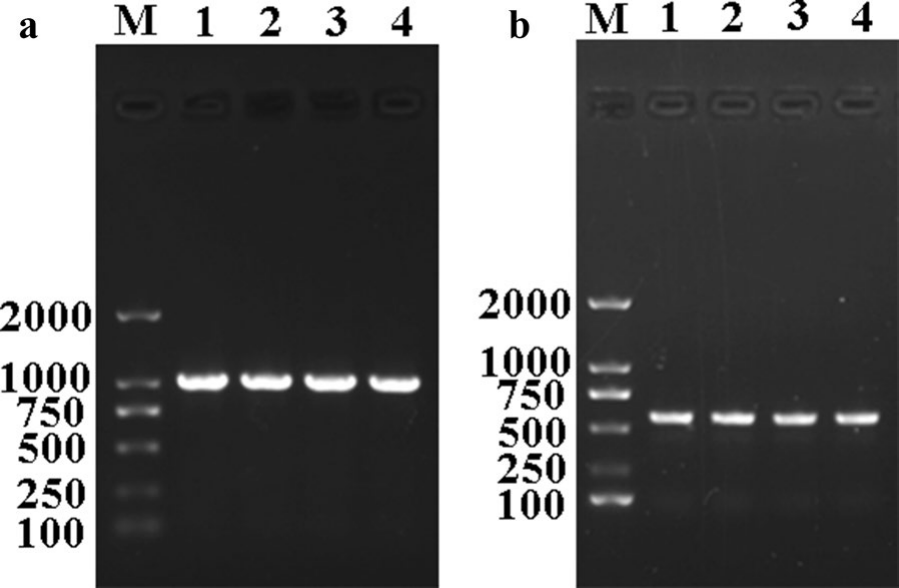
RT-PCR analysis of the transcription of TsCB (**a**) and GAPDH (**b**) for the life-cycle stages of *T. spiralis* studied. Lane M: DL2000 DNA marker; Lane 1: muscle larvae; Lane 2: IIL1 larvae; Lane 3: 3d adults; Lane 4: NBL

Transcription of the TsCB mRNA was assayed by RT-PCR for the four parasite life-cycle stages and the GAPDH gene was used as an internal control. A TsCB transcript (984 bp) was detected in muscle larvae, IIL1, adults and NBL. Primers for GAPDH also generated the expected size (570 bp) at all stages (Fig. 3).

### Western blot identification of rTsCB

**Fig. 4:**
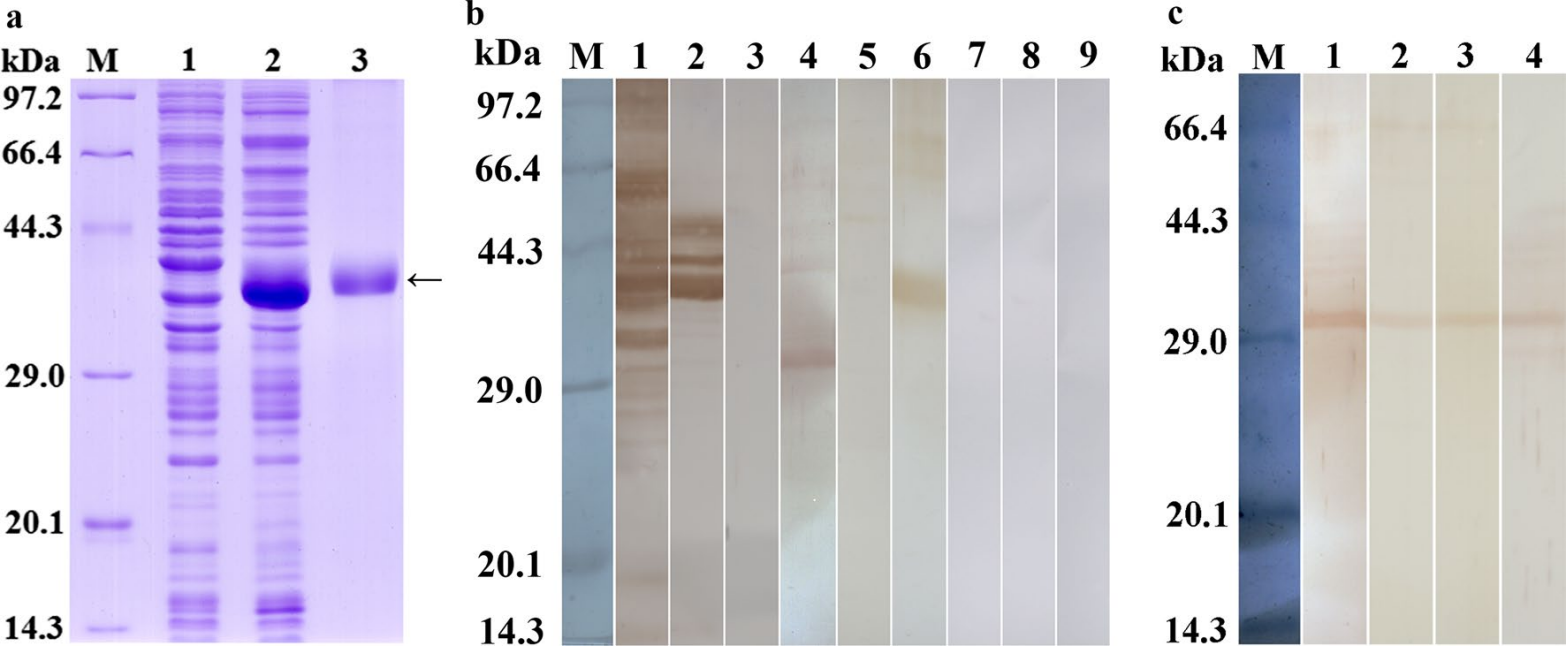
Identification of rTsCB. **a** SDS-PAGE analysis of rTsCB. Lane M: protein marker; Lane 1: lysates of recombinant *E. coli* carrying pQE-80L/TsCB prior to induction; Lane 2: lysates of recombinant *E. coli* carrying pQE-80L/TsCB following induction; Lane 3: purified rTSCB (arrow). **b** Western blot of rTsCB. Muscle larva somatic soluble protein (Lane 1) and ES protein (Lane 2) were recognized by mouse infection sera, but the rTsCB (Lane 3) was not recognized by infection sera. Native TsCB in muscle larva somatic protein (Lane 4) and rTsCB (Lane 6), not present in ES proteins (Lane 5), was detected by anti-TsCB serum. Muscle larva somatic protein (Lane 7), ES protein (Lane 8) and rTsCB (Lane 9) were not detected by normal mouse serum. **c.** Western blot showing that natural TsCB was detected using anti-TsCB serum in soluble somatic protein of the four life-cycle stages of *T. spiralis* (Lane 1: muscle larvae; Lane 2: IIL1; Lane 3: 3-day-old adults; Lane 4: NBL)

The results of SDS-PAGE revealed that the BL21 bacteria carrying PQE-80L/TsCB expressed a 39.7 kDa fusion protein. After purification, the rTsCB protein exhibited a clear individual band (Fig. 4a). The molecular weight (MW, 39.7 kDa) of rTsCB was identical to its predicted size.

Western blotting results exhibited that rTsCB was recognized by anti-rTsCB antibodies, but not by infection serum and normal mouse serum (Fig. 4b). Using anti-rTsCB antibodies, native TsCB was detected in soluble proteins of muscle larvae, IIL1, 3-days adults and NBL, but not in muscle larva ES proteins (Fig. 4b, c), indicating that the TsCB is one somatic protein of this nematode, but not a secretory protein from muscle larvae.

### Expression and localization of TsCB in the nematode life-cycle stages

**Fig. 5:**
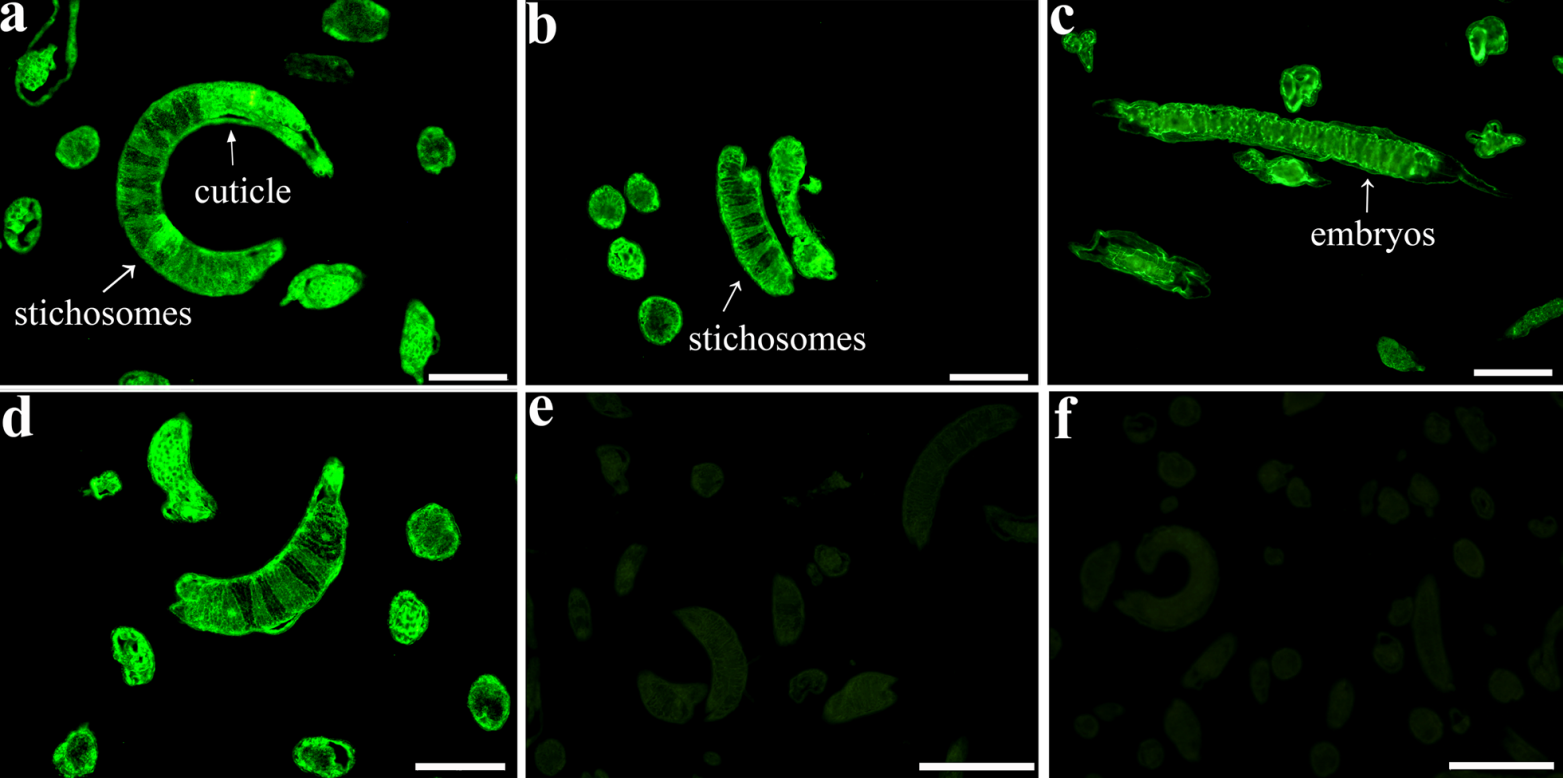
Location of TsCB of different life-cycle stages of *T. spiralis* by IIFA using anti-rTsCB antibody. Green fluorescence staining was detected in the cuticle and stichosome of the muscle larvae (**a**) and IIL1 larvae (**b**), 3-day-old females and embryos (**c**). Muscle larva incubated by infection sera was utilized as a positive control (**d**). Muscle larva incubated with pre-immune sera (**e**) and PBS (**f**) were used as negative controls. *Scale-bars*: 50 μm

The IIFA result revealed that the fluorescence staining was detected in the four life-cycle stages (ML, IL1, 3-days female adult and embryos) by anti-rTsCB antibodies. The fluorescence was distributed in the cuticle and stichosome of the nematode and in the embryos within female uterus (Fig. 5). Fluorescence staining with pre-immune serum was not observed.

### Specific antibody response

**Fig. 6:**
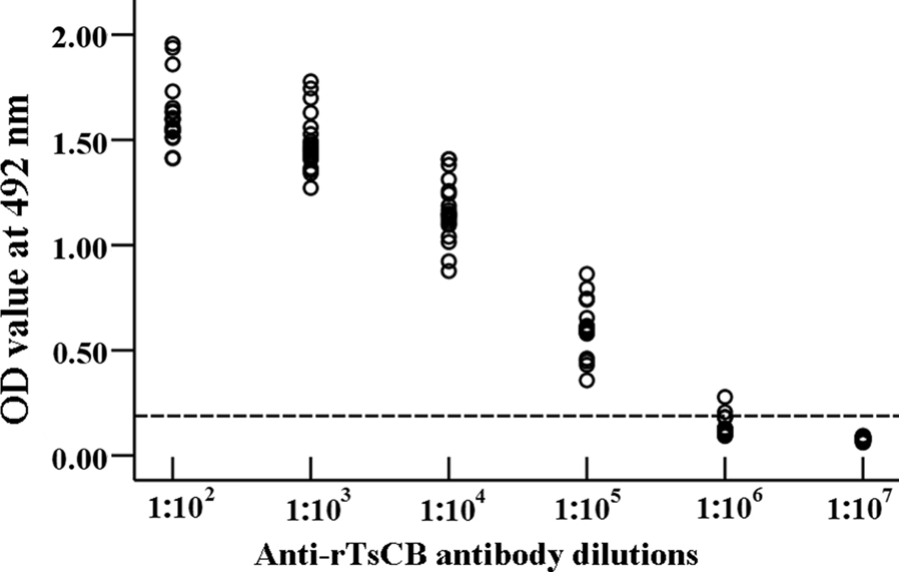
Serum anti-rTsCB IgG titers determined by rTsCB-ELISA. The OD values are shown as the mean ± SD of anti-rTsCB IgG levels of 20 immunized mice

**Fig. 7:**
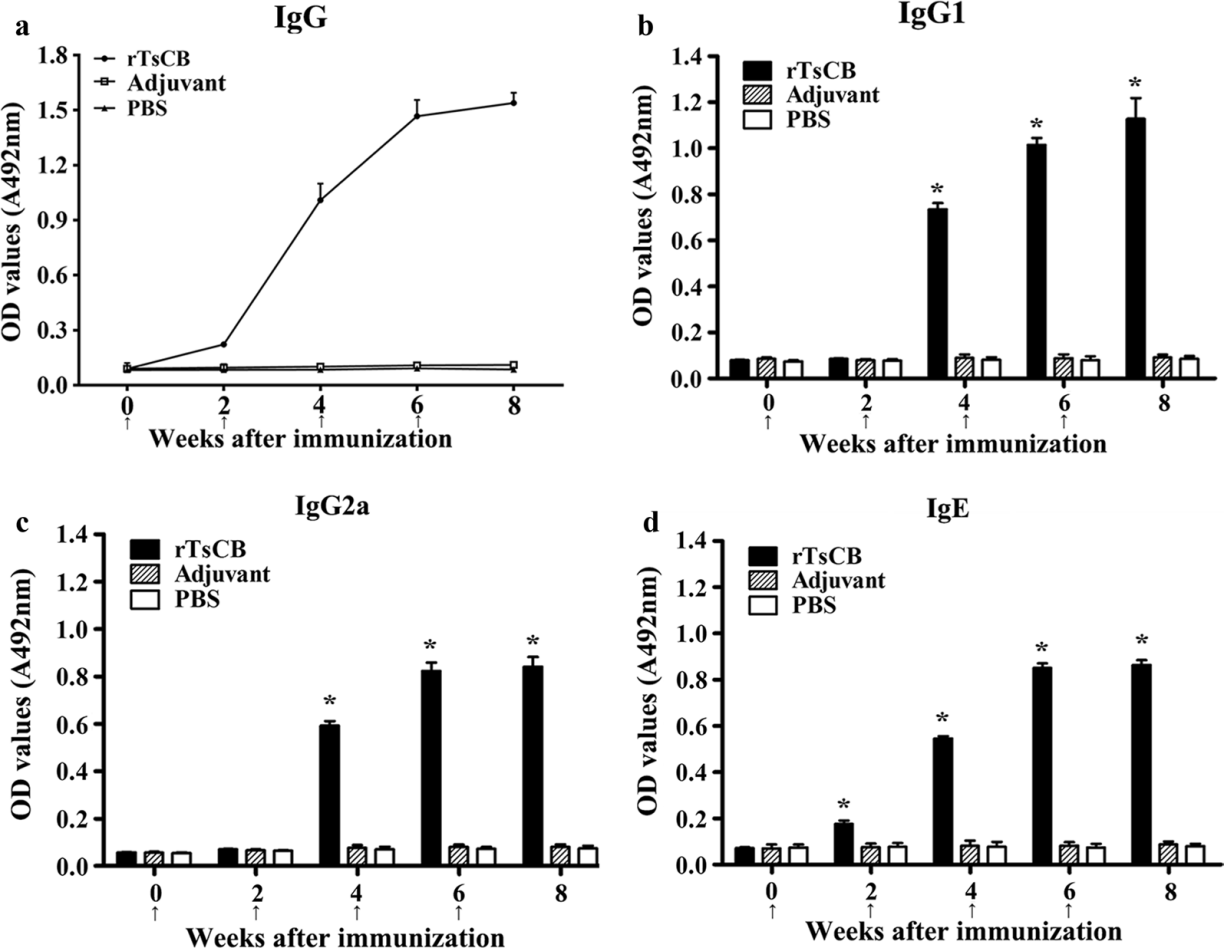
Specific antibody response in mice immunized with rTsCB. **a** Specific total IgG in mice immunized with rTsCB or control mice (adjuvant and PBS) at different time intervals following vaccination. Specific IgG1 (**b**) and IgG2a (**c**) subclass responses against rTsCB at different time points following vaccination. **d** Specific IgE level in vaccinated mice. The OD values are shown as the mean ± SD of antibody levels (*n* = 10). Vaccination time point is indicated with an arrow. *P < 0.001 compared with adjuvant or PBS group

To determine specific antibody responses to rTsCB, rTsCB-specific IgG, IgG1 as well IgG2a, and IgE in serum samples of vaccinated mice, responses were measured using an rTsCB-ELISA. The anti-rTsCB IgG titer was 1:10,000 after the third immunization (Fig. 6), indicating that the rTsCB was a strong immunogenic. The anti-rTsCB IgG level in vaccinated mice was prominently raised following the second immunization, whereas no mice vaccinated with ISA 201 or PBS exhibited any anti-rTsCB antibody responses (Fig. 7a). The IgG1 levels at 4, 6 and 8 weeks post-immunization were prominently higher than IgG2a (week 4: *t*_(18)_ = 4.350, *P *< 0.0001; week 6: *t*_(18)_ = 4.247, *P *< 0.0001; week 8: *t*_(18)_ = 2.902, *P* = 0.009) (Fig. 7b, c), demonstrating that immunization with rTsCB elicited a Th2-predominant Th1/Th2 mixed immune response. Moreover, anti-rTsCB IgE was also determined, and the results showed that the specific IgE level was significantly elevated in mice immunized with rTsCB in comparison to the control groups (*F*_(4, 45)_ = 568.102, *P *< 0.0001) (Fig. 7d), suggesting that specific IgE antibodies might play a crucial action in TsCB-induced rapid worm expulsion from the gut.

### Immune protection of rTsCB immunization against larval challenge

**Fig. 8:**
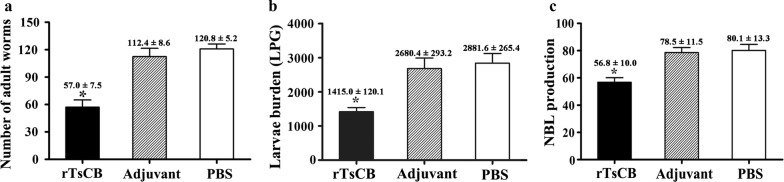
Protective efficacy of immunization with rTsCB against a challenge with 300 muscle larvae. **a** Number of adults in the intestine at 6 days post-infection. **b** Muscle larvae burden (larvae per gram of muscles, LPG) at 35 days post-infection. Worm burden is represented as the mean ± SD of 10 animals/group. **c** NBL production of each female adult from immunization and control mice. *P < 0.01 compared with adjuvant or PBS group

Compared with PBS control mice, the mice immunized with rTsCB exhibited a 52.81% reduction of intestinal adults at 6 dpi (Fig. 8a) and a 50.90% reduction of muscle larvae at 35 dpi (Fig. 8b) after oral challenge with 300 *T. spiralis* infective larvae. The *in vitro* NBL production for 72 h of adult females from rTsCB-immunized mice was significantly inferior to that of control mice (Fig. 8c) (*F*_(2, 27)_ = 11.153, *P *< 0.0001). This result showed that the immunization with rTsCB elicited an immune protection against the *T. spiralis* challenge infection.

**Fig. 9:**
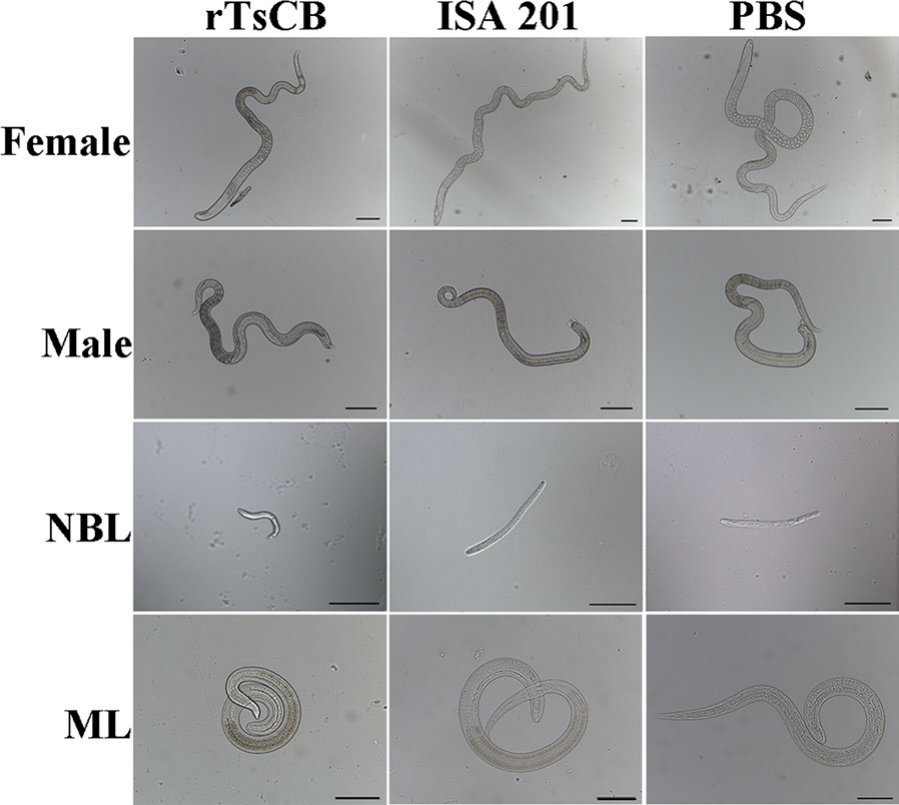
Morphology of various *T. spiralis* stages from mice immunized with rTsCB at 6 and 35 days following challenge with infective larvae. *Scale bars*: 100 μm

**Fig. 10:**
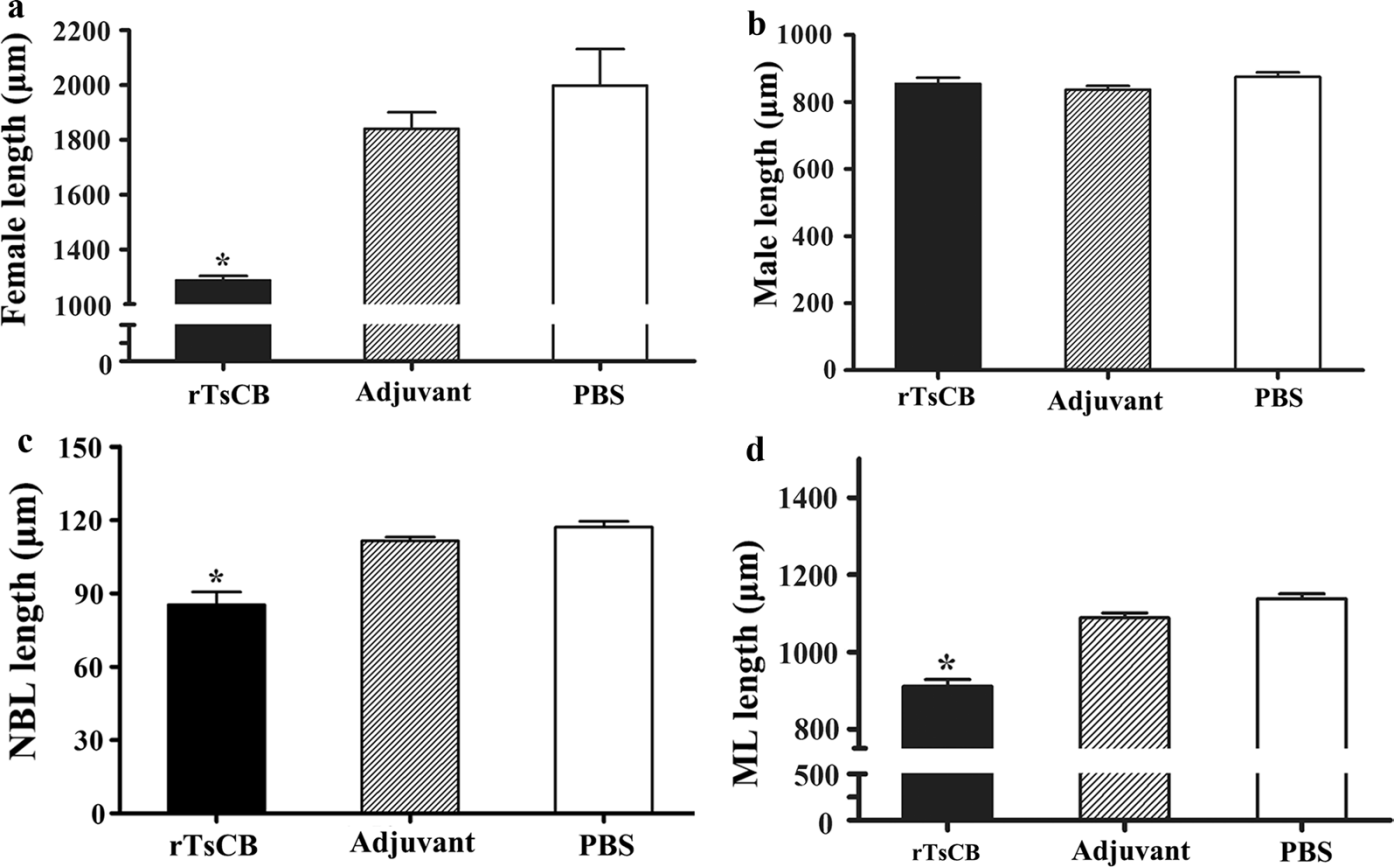
Length of different life-cycle stages of *T. spiralis* from mice immunized with rTsCB at 6 and 35 days after challenge (*n* = 10). **a** Female adult worms. **b** Male adult worms. **c** Newborn larvae (NBL). **d** Muscle larvae (ML). *P < 0.01 compared with adjuvant and PBS groups

The length of adult females collected from rTsCB-immunized mice at 6 dpi was evidently smaller than that from ISA 201 adjuvant or PBS control mice (Figs. 9, 10) (*F*_(2, 27)_ = 19.390*, P *< 0.0001); but the length of adult males did not show statistically significant difference among the three groups (*F*_(2, 27)_ = 1.849, *P* = 0.177). Moreover, the length of NBL produced by the adult females in immunized mice was clearly shorter than that from the PBS group (*F*_(2, 27)_ = 24.788, *P *< 0.0001). The muscle larva length from immunized mice was also significantly shorter than that of control mice (*F*_(2, 87)_ = 68.216, *P *< 0.0001). These results indicate that the immune response elicited by immunization with rTsCB also hampered the parasite growth and development, reduces the female reproductive capacity, as a result, alleviate the muscle larva burdens in immunized mice.

## Discussion

In the present study, the complete cDNA sequence of the TsCB gene was cloned, expressed, and its biological characteristics were investigated. The full-length TsCB sequence was 1071 bp encoding a 40.23 kDa protein. The amino acid sequences of the TsCB gene had 98% identity with the cathepsin B of six encapsulated species/gene types of the genus *Trichinella* (*T. nativa*, *T. murrelli*, T6, T8, *T. britovi* and *T. nelsoni*). Our results demonstrated that rTsCB were expressed in *E. coli*, with a molecular weight of approximately 40.23 kDa identical to the expected size. After being purified, rTsCB had strong immunogenic properties. Western blot results showed that native TsCB in somatic proteins of muscle larvae, IIL1, 3-day-old adults and NBL were identified by anti-rTsCB antibodies, but not in muscle larval ES protein, indicating that the TsCB is one somatic protein of this nematode, but not a secretory protein of muscle larvae. In the present study, TsCB transcription and expression were also investigated using RT-PCR and IIFA. RT-PCR results indicated that the TsCB gene was transcribed in the four *T. spiralis* life-cycle stages (muscle larva, IIL, adult and NBL). TsCB expression was detected by IIFA for all *T. spiralis* life-cycle stages, immunofluorescence staining was located in the cuticle, stichosome and intrauterine embryos of this parasitic nematode, suggesting that TsCB as a surface protein might play a role during larval intrusion of the host’s small intestinal epithelium [30, 58]. Surface proteins of *T. spiralis* intestinal stage worms are exposed directly to the host’s enteral milieu and local mucosal immune system, they are the important antigenic molecules, and can play a key role in larval intrusion and development [23, 24]. Previous studies have shown that recombinant *T. spiralis* surface proteins (nudix hydrolase, serine protease, cysteine protease, etc.) participate in larva penetration of intestinal epithelia [15, 19, 41]. Our previous study demonstrated that when the *in vitro* larva invasion experiment was performed, rTsCB promoted larva invasion of enterocytes, whereas rTsCB-specific antibodies suppressed larva invasion, this promotion or suppression was dose-dependent of rTsCB or rTsCB-specific antibodies. Silencing TsCB using RNAi significantly impeded the larva invasion (Han et al., unpublished data). The present study suggests that TsCB plays a major part on intestinal mucosal intrusion by this intracellular parasitic nematode.

Vaccination of mice with rTsCB elicited a specific Th2-predominant (higher level of IgG1) antibody response to rTsCB. The intestinal and muscle worm reduction observed in the present study is parallel with that of mice vaccinated with recombinant *T. spiralis* serine proteases [8], nudix hydrolase [45, 59] and glutathione S-transferase [42]. The immune protection induced by vaccination with rTsCB may be related to the generation of high levels of serum anti-TsCB IgG antibodies, which neutralized the capacity of cathepsin B to degrade enteral epithelium and other tissues of hosts [20]. Anti-*Trichinella* IgG may also bind to the epicuticle of enteral IIL1 larvae and generate an antigen-antibody complex in the larva anterior end, which may physically prevent parasite contact from intestinal epithelium cells, thus protect the intestinal epithelium from larval invasion [56, 60]. Antibodies against a cathepsin B-like protease (Ac-cathB-1) of *Angiostrongylus cantonensis* inhibited the L3 larva invasion of the intestines in rats [61]. In addition, previous studies indicated that anti-*Trichinella* IgG destroyed *T. spiralis* NBL and ML through an ADCC pattern [53, 62, 63].

In the present study, the level of anti-TsCB IgE serum in vaccinated mice was also determined. The results showed that vaccination with rTsCB elicited specific IgE, which plays a major role in the rapid expulsion of intestinal infective larvae and adult worms from the guts of vaccinated animals and in delaying larva invasion of intestinal epithelium after oral infection [64, 65]. Specific IgE is transported from the blood and exerts an active role in enteral lumen. The IgE combines with the worm surface of *T. spiralis* and mediates mast cell degranulation to prevent invasion [66, 67]. Moreover, IgE also plays an important action in destroying NBL by an antibody-dependent cellular cytotoxicity (ADCC) mode [68]. Our results demonstrated that vaccination with rTsCB elicited a high level of TsCB-specific IgG and IgE antibodies, which resulted in a significant reduction of worm burdens in the intestine and skeletal muscles of rTsCB-vaccinated mice. The results suggest that specific IgG and IgE antibodies are crucial for protective immunity against a *T. spiralis* challenge infection [69].

Additionally, our results also revealed that the length of female adults recovered from immunized mice and the female reproductive capacity (NBL production/female *in vitro* for 72 hours) was obviously lower than that of the ISA 201 adjuvant or PBS control mice. Length of NBL produced by females from immunized mice was significantly shorter than that of the ISA 201 and PBS groups. These results suggest that immune responses elicited by immunization with rTsCB also impeded intestinal worm growth, and declined the fecundity [56, 70]. A decline in female reproductive capacity might be related with females becoming shorter, since the uterus length has a correlation with female fecundity [71].

*Trichinella spiralis* is a multicellular parasite and its life-cycle is complicated. Different *T. spiralis* life-cycle stages have stage-specific antigens [72]. Vaccination with an individual *Trichinella* protein molecule only induced partial protective immunity against challenge. Therefore, oral polyvalent vaccines against diverse *T. spiralis* stages need to be developed [16, 44].

## Conclusions

TsCB was expressed in diverse life-cycle stages of *T. spiralis* and primarily located in the cuticle and stichosome of this intracellular parasite. Vaccination of mice with rTsCB elicited highly specific IgG and IgE responses and partial immune protection, as demonstrated by a significant worm burden reduction in the intestines and muscles of vaccinated mice after oral challenge with *T. spiralis* infective larvae. The humoral immune responses generated by immunization with rTsCB also impeded intestinal worm growth and declined its fecundity. The results show that TsCB might be considered as a novel potential molecular target to develop vaccines against *T. spiralis* infection.

## Data Availability

The data supporting the conclusions of this article are included within the article.

## Abbreviations

ADCC: antibody-dependent cellular cytotoxicity
AW: adult worms
DAB: 3,3′-diaminobenzidine tetrahydrochloride
dpi: days post-infection
ES: excretion/secretion
GAPDH: glyceraldehyde-3-phosphate dehydrogenase
IIFA: indirect immunofluorescence assay
IIL1: intestinal infective L1 larvae
ML: muscle larvae
MP: maximum parsimony
NBL: newborn larvae
rTsCB: recombinant TsCB
TsCB: *Trichinella spiralis* cathepsin B
